# Challenges in the association of human single nucleotide polymorphism mentions with unique database identifiers

**DOI:** 10.1186/1471-2105-12-S4-S4

**Published:** 2011-07-05

**Authors:** Philippe E Thomas, Roman Klinger, Laura I Furlong, Martin Hofmann-Apitius, Christoph M Friedrich

**Affiliations:** 1 Fraunhofer Institute for Algorithms and Scientific Computing (SCAI), Department of Bioinformatics, Schloss Birlinghoven, 53754 Sankt Augustin, Germany; 2 Knowledge Management in Bioinformatics, Humboldt-University Berlin, Unter den Linden 6, 10099 Berlin, Germany; 3 Research Unit on Biomedical Informatics (GRIB), IMIM-Hospital del Mar, UPF, PRBB, c/Dr. Aiguader 88, E-08003 Barcelona, Spain; 4 University of Applied Science and Arts Dortmund, Department of Computer Science, Emil-Figge-Str. 42, 44227 Dortmund, Germany

## Abstract

**Background:**

Most information on genomic variations and their associations with phenotypes are covered exclusively in scientific publications rather than in structured databases. These texts commonly describe variations using natural language; database identifiers are seldom mentioned. This complicates the retrieval of variations, associated articles, as well as information extraction, *e. g.* the search for biological implications. To overcome these challenges, procedures to map textual mentions of variations to database identifiers need to be developed.

**Results:**

This article describes a workflow for normalization of variation mentions, *i.e.* the association of them to unique database identifiers. Common pitfalls in the interpretation of single nucleotide polymorphism (SNP) mentions are highlighted and discussed. The developed normalization procedure achieves a precision of 98.1 % and a recall of 67.5% for unambiguous association of variation mentions with dbSNP identifiers on a text corpus based on 296 MEDLINE abstracts containing 527 mentions of SNPs.

The annotated corpus is freely available at http://www.scai.fraunhofer.de/snp-normalization-corpus.html.

**Conclusions:**

Comparable approaches usually focus on variations mentioned on the protein sequence and neglect problems for other SNP mentions. The results presented here indicate that normalizing SNPs described on DNA level is more difficult than the normalization of SNPs described on protein level. The challenges associated with normalization are exemplified with ambiguities and errors, which occur in this corpus.

## Introduction

Sequence variations are changes of the genetic material, usually DNA, of an organism. They are important to increase the variance of the genetic pool of species but may also lead to severe hereditary diseases like Huntington disease, Cystic fibrosis or Hemophilia. Two terms are commonly distinguished when referring to variations on the DNA level: mutation and polymorphism. Polymorphism are alterations with a minor allele frequency of ≥ 1 % in a particular population. Variations with a lower frequency are usually called mutation. However, the term mutation is also often used to imply a deleterious effect of a sequence variation without any knowledge about the underlying frequency distribution. Throughout this publication we use the term *variation* to describe arbitrary changes in a genomic sequence while *variation mention* refers to the textual description of a variation. Differences in a single nucleotide between members of one species are referred to as single nucleotide polymorphism (SNP). SNPs are a subclass of sequence variations, encompassing single base exchanges, single base deletions and single base insertions. It is assumed that 90 % of all human sequence variants are SNPs [1] and that they occur in average about every 100 to 300 bases [2,3]. SNPs are, therefore, the major source of human genetic heterogeneity. Diseases like Sickle–cell anemia, β Thalassemia or Cystic fibrosis might result from a SNP [4-6]. Some SNPs are associated with the metabolism of different drugs [7-9] and are, therefore, relevant for research areas like pharmacogenomics. SNPs without an observable impact on the phenotype are still useful as genetic markers in genome wide association studies, because of their sheer quantity and the stable inheritance over generations.

Information on SNPs is covered in curated databases. Nevertheless, the wealth of information about the clinical impact of SNPs is contained in free text in the form of biomedical publications. At the moment, PubMed provides access to more than 19 million citations contained in MEDLINE. The described SNP mentions need to be interpreted to be valuable, either by a human curator alone or supported by a text mining system. This interpretation often requires the normalization of the SNP mention. By normalization we refer to the association of SNP mentions in text with their corresponding database identifiers, for instance from a sequence database such as dbSNP.

The interpretation of SNP mentions is challenging due to ambiguous use of different nomenclatures, missing information in a publication or sloppiness in the description. Automated text mining methods are able to extract SNP mentions from text, but only few associate these with unique identifiers in SNP databases.

The main contribution of this paper is the description and analysis of these challenges and to provide background knowledge to either build such a system or to interpret SNP mentions in text. The paper is organized as follows: A brief summary of different SNP data sources is given in Section *SNP Data Sources*, followed by a description of different problems of finding a database identifier for a SNP mention in Section *Normalization Process*. This section reviews the evolution of a human mutation nomenclature, common problems in named entity recognition, provenance and other problems. Subsequently previous approaches for automated extraction of variation mentions are discussed. The generation of a corpus and the implementation of our normalization algorithm is described in Section *Methods* and the relevancy of error types is estimated on this corpus in Section *Results and Discussion*.

## SNP data sources

Detailed information about SNPs can be found in various databases like Online Mendelian Inheritance (OMIM) [10], jSNP [11], or the Human Gene Mutation Database [12]. OMIM focuses on the relationship between phenotype and genotype and cites the corresponding publications while jSNP is a repository of Japanese SNP data and the Human Gene Mutation Database constitutes a collection of data on germ-line mutations.

All these databases have links to the dbSNP database [13], which is the most comprehensive resource with 55 organisms and more than 63 million unique SNP entries. Every single entry is accessible via a unique database identifier called refSNP or “rs number”. The content of dbSNP is interconnected with many other resources, *e*. *g*. EntrezGene [14], GenBank [15], the Universal Protein Resource (UniProt) [16], HapMap [17], Ensembl [18] or SNPedia [19].

**Figure 1:**
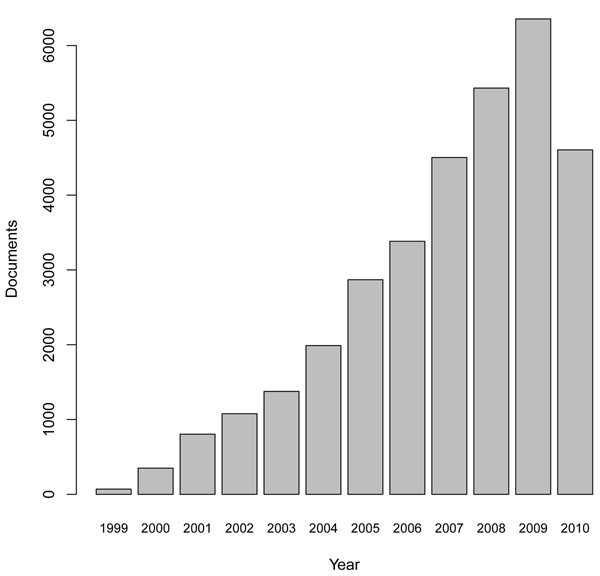
Number of articles annotated with MeSH Term: “polymorphism, single nucleotide”.

All these different sources contain valuable information like primer sequence, population frequency or information on the corresponding gene, but only little about the biomedical implication. This information is mostly covered in publications, which are stored in databases like MEDLINE. The National Center for Biotechnology Information (NCBI) provides references to 4487 articles for 24079 dbSNP entries. Due to high-throughput techniques like SNP-arrays [20], mass spectrometry [21,22], and new DNA sequencing methods [23,24], the amount of SNP related data and publications is rapidly increasing. The number of articles annotated with MeSH term “polymorphism, single nucleotide” is depicted in Figure 1.

In the following, we describe characteristics regarding the process of finding database identifiers for free text mentions of human SNPs. More precisely, this work focuses on SNP substitutions in Homo sapiens with about 18 million entries (as of dbSNP version 128).

## Normalization process

### Workflow

**Figure 2:**
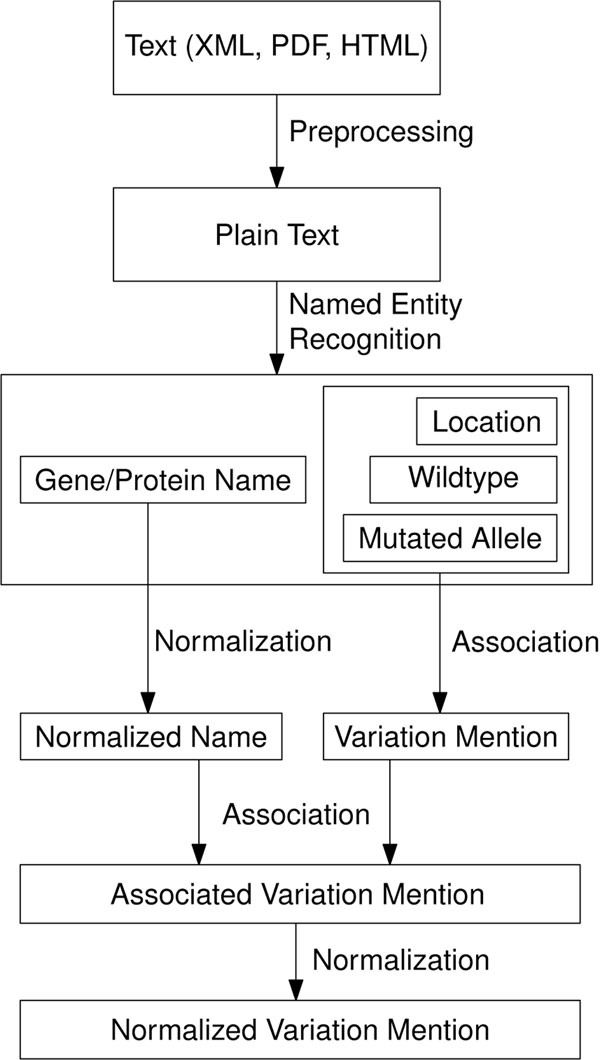
Representative workflow for extracting SNP information from unstructured text.

A general workflow for the automated extraction of SNP mentions from literature is illustrated in Figure 2. The figure shows required subtasks for the extraction and subsequent normalization of SNP mentions. The individual tasks are subsequently described and task specific problems are highlighted. For the description of the concrete implementation we refer to the *Methods* section.

In contrast to a human who typically perceives the provided document in the published form best (*e. g.* hard copy, pdf, html), an automated machinery needs a uniform text representation that necessitates a preprocessing step (conversion of XML formats or extraction of plain text from full text documents). The mentions of SNPs in different nomenclatures or natural language need to be detected as well as the gene names. While this task is typically easily accomplished by a human, it is challenging for an automated system due to the huge amount of different complex formulations found in free text.

Based on the detected SNP mention and the gene/protein names (and their normalization to the databases like EntrezGene or UniProt) and their association, the normalization process is performed. This process is straight-forward for direct mentions of identifiers but highly problematic for other formulations.

The aim of SNP normalization is to correctly associate SNP mentions in text with unambiguous database identifiers. Thus, it is necessary to understand how these entities are typically described. To normalize a SNP the *wild type*, *mutated allele*, and *location* on the reference sequence is required. Further the underlying *gene* or *protein* needs to be identified. Whereas the terms wild type and mutated allele describe biological concepts, the rules to determine the position of a variation on a certain gene have changed recently. The following subsection describes the most important changes in the nomenclature for human mutations.

### Human mutation nomenclatures

This section briefly summarizes some concepts of human mutation nomenclature and its evolution relevant for the normalization of SNP mentions. Prior to the first recommendation of a common nomenclature many different descriptions were arbitrarily used. For some alleles nicknames like “hemoglobin Crete” [25], “haemoglobin Guantanamo” [26], “Factor IX Angers” [27], “Factor IX Bordeaux” [27], or “α_1_-antitrypsin Pittsburgh” [28] were commonly used. As articles became harder to interpret by non domain experts, in 1993 the emerging problem of many different nomenclatures lead to first initiatives to define a nomenclature covering different types of genetic variations.

Genetic sequence changes occur in general on the DNA level. Variants located on an exon may be propagated to mRNA and may consequently lead to a change of the encoded polypeptide chain. Therefore, a SNP can be described on at least one of these three levels. However, SNPs are usually described on protein or DNA level. To distinguish between these two concepts, we use the terms protein sequence mutations (PSM) and nucleotide sequence mutations (NSM) [29]. For instance, the first sentence of the publication from Wolff et al. [30] describes the same SNP as NSM (894G– –>T) and PSM (Glu(298)– –>Asp and E298D):

“The Glu(298)– –>Asp (E298D; 894G– –>T) polymorphism of eNOS (endothelial nitric oxide synthase) has been related with cardiovascular disease.”

All three SNP mentions can be further associated with the unambiguous dbSNP identifier rs1799983. For simplicity, the following sections are focusing on SNPs, but can be applied to many other types of variations analogously. However, the description of complex variations, like changes within duplications, can become rather complicated.

#### First recommendation for mutation mentions from 1993

In the first publications on this topic the idea that positions of PSM should always be deduced from the primary translation product was introduced [31,32]. This seems reasonable, but previously described PSMs were often deduced from mature proteins or cleavage products [33-35]. The first amino acid of the primary translation product, in human usually a methionine, is defined as position +1. Earlier publications often started to count one position after the initiator methionine, because it is frequently cleaved. For example, the sickle cell causing allele located on hemoglobin–β (HBB) is usually referred to as a replacement of a glutamatic acid by a valine at position 6, or commonly abbreviated Glu6Val or E6V. However, the corresponding dbSNP entry rs334 reveals that the polymorphism is located on position 7. Additional examples of PSMs with a position shift of 1 in the HBB locus can be found at the corresponding OMIM entry in the category “Allelic Variants”.

**Figure 3:**
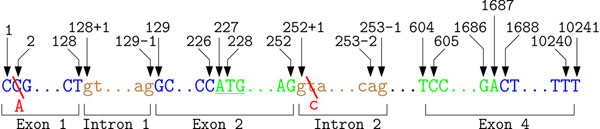
**Illustration of the first recommendation for a common mutation nomenclature.** Example annotation for parts of gene MECP2 (GenBank entry: NG_007107.1) using the first suggestions for a mutation nomenclature. Exonic sequence is labeled green, intronic regions are labeled brown and the surrounding untranslated regions are labeled in blue. In the first suggestions for a common nomenclature the most 5’ sequence of the first exon is the start position. Adjacent bases are subsequently numbered. Variations occurring in intronic regions obtain two numbers. The first describes the location of the closest exon and the second is the distance to this exons. As shown in this picture, intronic positions are usually described in relation to the closer exon. The underlined ATG marks the start codon, where the leading adenine has been later proposed as common start position. Using these recommendations the two SNPs are described as 2C→A and 252+2T→C

Consequently several suggestions to describe NSM have been made. In contrast to PSM, no intuitive start position exists. The first approach suggested to use the exact 5’ cap site as position +1. If the exact cap site is unknown, the most upstream known cDNA base is used as start point. When publishing these recommendations in 1993 and 1996 respectively, only few complete cDNA sequences were available and the human genome project was in its infancy. Therefore, it has been concluded that this system leads to an arbitrary numbering based on early sequence data. Bases upstream of the cap site should be consecutively numbered as –1, –2, –3 and so on. Numbering downstream follows the cDNA sequence, meaning that only exonic regions are consecutively numbered. Bases within an intronic region are described by two numbers. The first number is the base of the closest exon and the second is the relative distance to this base. This convention allows to describe intronic variants, without even knowing the exact length of the intronic region. The recommendation to describe NSMs on gene MECP2 using this first nomenclature are depicted in Figure 3. To trace the changes in mutation nomenclature two persistent examples are introduced and labeled in the corresponding figures. Using the described recommendations the first SNP is referred to as 2C→A and the intronic SNP is described as 252+2T→C.

#### Change of start position counting for nucleotide sequence mutations in 1996

**Figure 4:**
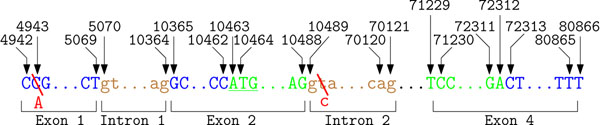
**Illustration of the recommendations using the genomic sequence.** The example annotations for parts of the gene MECP2 (NG_007107.1) are following the genomic DNA numbering concept. Numbering starts at the beginning of the used reference sequence. Following bases are consecutively numbered. Using these recommendations the two SNPs are described as 4943C→A and 10490T→C

Numbering based on genomic DNA is the most robust form of systematic nomenclature [36,37]. In this case, the DNA bases are consecutively numbered from a common start position. However, when proposing these recommendations in 1996 and 1998 the full genomic sequence was not available for all genes. The positions of SNPs for MECP2 using genomic DNA are depicted in Figure 4. Using this recommendation the two SNPs are described as 4943C→A and 10490T→C.

**Figure 5:**
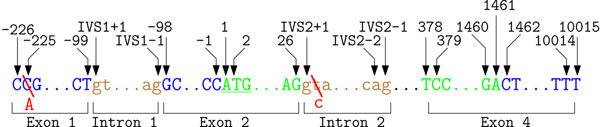
**Illustration of the “intervening sequence” concept in human mutation nomenclature.** The example annotations for parts of the gene MECP2 (NG_007107.1) are following the IVS concept. In this nomenclature variant the adenine of the start codon is used as start position. Variations located in intronic regions start with the abbreviation “IVS” followed by the number of the intron where the variation is located. The consecutive number determines the distance to the next intron/exon boundary. Using these recommendations the two SNPs are described as –225C→A and IVS2+2T→C

Antonarakis as well as Beutler et al. [36,37] suggested to use cDNA if no reliable genomic DNA sequence is available. Instead of the exact cap site, both publications recommended to use the adenine of the initiator ATG site as common start position. To avoid confusion regarding the type of used reference sequence, the variation mention is preceded by “g.” for genomic or by “c.” for cDNA. The accession number for the used primary sequence database should be mentioned in the text. SNPs occurring in introns have to start with the abbreviation IVS (intervening sequence) followed by the number of the intron where the variation occurs. The following number determines the distance to the closest exon. The derivation of positions for NSM on MECP2 using the described changes is depicted in Figure 5. Using this nomenclature, our example SNPs are described as -225C→A and IVS2+2T→C.

#### Explicit statement of variation level in 2000

**Figure 6:**
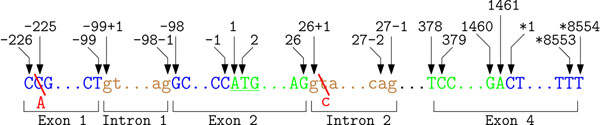
**Illustration of the latest recommendations for human mutation nomenclature.** This most recent nomenclature discards the IVS concept for intronic variations. Instead, the concept introduced earlier using two numbers is again recommended. Variations occurring in the 3’UTR are labeled with a preceding asterisk and numbering starts at the beginning of the UTR. Using these recommendations the two SNPs are described as c.–225C>A and c.26+2T>C

The nomenclature updates [38,39] introduced new concepts to cover more complex sequence variations and to reduce ambiguities. Every variation has to begin with a single letter indicating the type of reference sequence (genomic DNA, cDNA, mitochondrial DNA, RNA, or protein) of the described variation. For example, the description r.67g>u specifies that mRNA is used as reference sequence for this SNP. The nomenclature allows no variation in the textual description of variation mentions, which allows to extract them easily *e. g*. by regular expressions. Variations occurring in the 3’UTR are designated with a preceding asterisk (*) and the distance to the last base of the stop codon. Applying this nomenclature to our example variations results in c.–225C>A and c.26+2T>C. A visualization of the new recommendations applied on MECP2 is depicted in Figure 6.

#### Update for intronic variations in 2007

In the most recent publication [40] rules introduced in [38,39] were recapitulated. The IVS concept for intronic variations has been replaced by the previously introduced idea, that intronic SNPs are described by two numbers. The first number presents the location of the closest exonic base and the second number describes the relative distance to this base. Every variation may start with the accession number of the used reference sequence followed by a colon and the description of the variation (*e. g.* NM_005957.3:c.123G>T). These variation mentions can be easily extracted by using regular expression. The explicit statement of the used reference sequence facilitates the normalization to a unique identifier. Our running examples are now depicted as NG_007107.1(MECP2):c.–225C>A and NG_007107.1(MECP2):c.26+2T>C. In these two examples the gene is mentioned in brackets, because the used reference sequence NG_007107.1 covers two genes (MECP2 and IRAK1). The direct mention of the gene of interest in the expression is required to avoid ambiguity. For instance, the description NG_007107.1:c.1A>T may refer to a substitution on both genes. An increasing number of journals, like *Human Mutation*, insists on using the latest recommendations for a common human mutation nomenclature. The increasing complexity of guidelines makes the description of newly discovered mutations an error prone and time intensive process. Tools like Mutalyzer [41] assist the creation and validation of a valid description of sequence variations and may help to reduce human errors. For example, Mutalyzer converts genomic coordinates to transcript orientated positions and allows to validate the correct description of a submitted variation mention. Another application useful for the conversion of different SNP description is SNP-converter [42]. More and more publications describe SNPs also in terms of dbSNP accession numbers [43], which is supported by the latest mutation nomenclature. For example, the mention rs2306220:A>G is a valid SNP description.

For NSMs an intuitive but important issue is that human genes often have more than one transcript variant and every transcript has its own unique exon/intron boundaries and start codon. Usually a NSM can be described with respect to all associated reference sequences. However, the description of a mutation may differ between two reference sequences due to the unique properties of a reference sequence. Explicitly mentioning the used reference sequence including a version number avoids this problem and is, therefore, recommended.

#### Recapitulation

**Figure 7:**
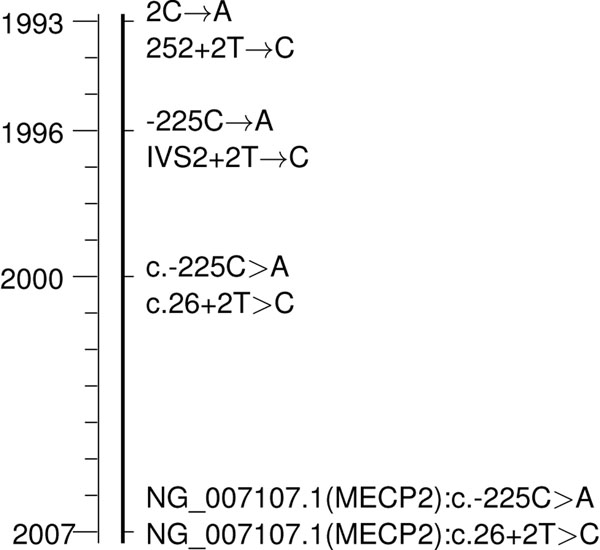
Proposed spellings for mutations over the last years.

The guidelines to describe variations have been recently changed [36-40]. For example the previously introduced NSMs can be reported in several ways, depending on the year of publication. The different notation variants are depicted in Figure 7. It is noteworthy that the use of a specific nomenclature does not necessarily imply that each mutation can be unambiguously described. For example intronic variations can be described on cDNA reference sequence with respect to the 5’ or 3’ exon. However, a common and unambiguous nomenclature is important to reduce errors. For example, due to inconsistencies in notations, the distinct mutation c.439 443delGAAGT has been individually reported by two research groups as different mutation (425del5 and 472del5) [44]. Additionally, various human errors have been reported. Amino acids sharing the same initial letter (*e. g.* Alanine, Arginine, Asparagine, and Aspartic acid or Threonine, Tryptophan and Tyrosine) are often wrongly abbreviated when using one letter abbreviations [38]. Also counting errors in the description of mutations have been reported [44].

Even though the rules to calculate the position of a PSM remained unchanged since the first publication, two commonly used numbering variants have been reported:

First, PSMs may be deduced from the mature protein instead of the precursor polypeptide. This is the case for the protein Lymphotoxin-alpha (LTA), where the precursor peptide contains a signaling sequence from position 1 to 34. After transport of LTA to the final destination, the signaling peptide is cleaved by a signal peptidase. This is the reason why some authors refer to position 35 as 1 and vice versa. This specific information is covered in the feature table of the corresponding UniProtKB entry P01374. This property has been described and used by Yip et al. [34] to successfully normalize PSMs deduced from the mature protein sequences. The authors found that relevant information is covered in the features *Signal*, *Transit*, *Peptide*, *Propeptide* and, *Var_seq* of the corresponding UniProt entry.

The second problem is that some publications start to count one position after the leading amino acid. Therefore, some dbSNP entries have an offset of +1 compared to the textual description. This has been previously exemplified for the sickle cell allele Glu7Val located on Hemoglobin beta (HBB). Evidence for cleavage of the first amino acid can be found in the feature *Initiator methionine* of the respective UniProt entry P68871.

### Identification and association of gene and variation entities

#### Challenges in named entity recognition

For the normalization of SNP mentions to sequence database identifiers, detection of SNP mention and the associated gene or protein names is crucial. For automated recognition, several tools have been proposed, both for gene and protein names [45-51] and variation mentions [29,33,34,52,53]. For details, we refer to the original publications. Recognizing biological terms is often mislead by the lack of a commonly accepted nomenclature. Therefore, the problem of word sense disambiguation and abbreviation disambiguation has to be handled. Typical examples of words representing a PSM and another biological concept are exemplified in Table 1. The frequent occurrences of some of these terms in MEDLINE highlight the relevancy of an elaborate disambiguation system. An example for homonymous gene names has been exemplified by Weeber et al. [54] for the abbreviation PSA. PSA is a valid gene identifier for prostate specific antigen (GeneID 354), puromycin-sensitive aminopeptidase (GeneID 9520), protein S, alpha (GeneID 5627) and phosphoserine aminotransferase (GeneID 29968). Additionally, the abbreviation corresponds to other concepts like psoriasis arthritis, poultry science administration, pig serum albumin or psoriatic arthritis. The performance of different text mining systems for gene mention recognition and for gene normalization to database entries has been critically assessed in BioCreAtIvE I [55] and II [56].

**Table 1:** Examples of mentions which may refer to a variation or a different concept.

Abbreviation	Alternative concept	Frequency
C3H	Zinc finger protein ZF	25,241
E2F	Transcription Factor	11,796
H2S	Hydrogen sulfide H_2_S	3,726
T47D	Breast cancer cell line	2,902
L5178Y	L5178Y mouse lymphoma cells	2,736
T1D	Type 1 diabetes	2,731
T98G	Human glioblastoma cell line	1,244
H295R	Adrenocortical carcinoma cell line	637
P4501A	Cytochrome P4501A	485

Frequency is the occurrence of abbreviations over all MEDLINE abstracts. Query performed 2009/01/21.

#### Association of SNP sub-entities

**Figure 8:**
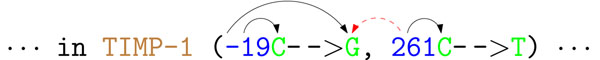
**Exemplified depiction of a paragraph annotated by a machine learning tool.** Prior to normalization all sub-entities (alleles and location) have to be combined into tuples of entities. In this example the location 261 can be wrongly associated with the two closest states G and C. This can be circumvented by punishment of punctuation marks between two entities, like the comma in this case.

For the normalization of SNP mentions, the location, wild type and mutated allele have to be known. Approaches relying on machine learning methods commonly identify wildtype, mutated allele and location separately [43,57]. These approaches require subsequent association with extracted sub-entities (alleles and location) to build a complete SNP tuple. A descriptive example is given in Figure 8, where two different NSMs are identified. SNP tuples could be created by associating each location with the two closest alleles. In our example this would create the triples (–19,C,G) and (G,261,C), where the second triple would be wrong. To circumvent this problem it may be useful to regard if the preceding or succeeding word is a delimiting character like a comma, bracket, or dot.

However, the creation of these tuples introduces a new error source, but the use of machine learning approaches to identify variation mentions in text has a substantial advantage: Approaches relying on regular expressions commonly extract only diallelic variations like L69K or 32C– –>T. However, for human triallelic SNPs (rs3091244), tetraallelic SNPs (rs293806), or even pentaallelic SNPs (rs1049092) have been observed. SNPs like 82C– –>T/G are currently only extracted by machine learning tools. Some authors describe only the observed allele of a variation like “52L” or the genotype like “–403 AT” or “–403 AA”. Even though these examples are not mutations, it is feasible to detect and normalize such mentions. Approaches detecting alleles and location separately provide more flexibility but the subsequent association introduces an additional error source.

#### Extraction of dbSNP identifiers

One kind of SNP mention found in biomedical text is the direct citation of a dbSNP identifier. In such cases, the SNP can be unambiguously identified and mapped to dbSNP. In a previous experiment [43], such mentions have been extracted with the regular expression “[rR][sS][ ]*[1–9][0–9]*”. On a test set of 300 extracted mentions, a recall of 100 % and a comparatively small precision of 74 % has been achieved. Similar to other named entity recognition tasks, some extracted mentions describe a different concept. A list of observed false positives can be found in Table 2. The regular expression has been improved by accepting capital rs mentions only, when keywords like “mutation” or “SNP” matched and exclusion words like “strain” did not occur in the abstract. The precision has been further improved using a blacklist with recent false positives like rs61443. The resulting tagger reached in a sub-sampling of 300 mentions a precision of 97 % at an approximate recall of 98 %. In the same publication it has been shown, that direct mention of rs numbers in MEDLINE abstracts increases steadily since 2002.

**Table 2:** Examples for potentially wrong extracted dbSNP identifiers using the naive regular expression [rR][sS][ ]*[1–9][0–9]* interfering with different concepts [43].

rs number	Alternative concept
rs1	Cell Line
rs6000	Computer Name
rs485	Computer Interface
rs1000	Indian Rupees
rs61433	Immune Suppressor

#### Gene-SNP association

Prior to a normalization each detected SNP mention has to be correctly associated with the corresponding gene. This is necessary because the location of a SNP is described in relation to the corresponding gene. This is a difficult task even when only one single gene or protein is described in the text, because it is not guaranteed that this gene or protein is mentioned together with the SNP mentions in the text.

Several methods for the association of gene or protein with the corresponding SNP have been proposed. Some consider sentence boundaries [29], while other approaches associate a SNP with all extracted genes and try to validate the results using the dbSNP database [43,53]. Another approach introduced the “graph bigram association algorithm” for the purpose of building the required protein-variation pairs [58]. The algorithm removes all stop words from a text, builds a list of bigrams and calculates the likelihood that two words occur adjacent to each other using the t-statistic. Regarding only articles with more than one possible protein association the precision of the relation extraction using graph bigram is 84 % whereas the precision of the word distance measure is 73 %.

### Provenance

Genomic information rapidly changes over time. This also includes the sequence of the genome and its annotation. In 2004 the sequence of the human genome covered already 99 % of the euchromatin sequence with an error rate of 0.01 ‰ [59]. However, this sequence consisted of 308 gaps on euchromatic material in regions which are hard to sequence. Therefore, it is not surprising that the sequence of the genome underwent some updates since 2004. Without additional information, the human genome is just a long concatenation of characters using a comparatively small alphabet (A,T,G,C). Annotation information of the genomic sequence is needed to put meaning to this heap of data. Genes are often annotated using evidence like protein or cDNA sequences. Due to additional evidence or a change in the genome assembly the structural annotation of genes may alter. The change of annotated transcripts in the ENSEMBL database between version V46 and V47 has been investigated by the authors. Between these two versions the algorithm for transcript and UTR placement has been changed and therefore many changes can be expected. Approximately 33 % (12,435/38,238) of all shared transcripts had a different location for the start codon. NSMs located on one of these transcripts would obtain new position numbers.

Problems of not being able to normalize a SNP mention because of database changes are hard to solve. Changes in the reference sequence as well as merging or renaming of identifiers are often not well documented. To circumvent these problems, the latest human mutation nomenclature advises authors to mention the accession number of the used reference sequence in front of the variation description. Adherence of this rule simplifies the normalization substantially, because the respective reference sequence is known.

It has been previously mentioned by Antonarakis et al. [37], that the accession number of the used reference sequence should be additionally included in the publication. However, in some publications the accession number is not mentioned in the abstract. Therefore, it may be beneficial to incorporate the most appropriate sequence based on the publication date of an article. It is also crucial to use a SNP database which is derived from the same genome build as the used sequence database. Otherwise the position of a SNP can be miscalculated. Only the most recent version of dbSNP is available for download. However, the mapping information to the previous build is available.

### Allele information in dbSNP

A SNP is always observable on both strands because of the structure of DNA. Therefore, the alleles can be described on any strand side. For example, the replacement of an adenine by a cytosine on one strand leads to a replacement of a thymine by a guanine on the anti-parallel strand. To avoid confusion in the textual description of variations, alleles are always described on the same strand as the reference sequence and cDNA sequences are usually on the same strand as the associated gene.

In contrast, alleles in dbSNP may be arbitrarily located on any strand side. This is based on the build process of dbSNP. Submitted sequences obtain a unique and stable submission sequence (ss) number. The submitted sequence is aligned to the genome in question. Submission sequences describing the same SNP are merged into one single rs entry. A new rs entry is generated, if no ss previously covered this specific sequence variation. Regardless of the number of ss entries, every rs cluster has exactly one reference sequence. The reference sequence is always the longest submission sequence of each cluster and may be arbitrarily located on the sense or antisense strand. Therefore, the alleles are, depending on the alignment of the sequence to the genome, located on either strand. Due to this property, some alleles of dbSNP are on the opposite strand than the cDNA sequence. Information about the placement of a SNP on the current contig can be found online in the database table SNPContigLoc. Additionally information about the placement of a contig on the chromosome is contained in table ContigInfo.

### Ambiguity between PSM and NSM

The shared alphabet between protein and nucleotide sequences introduces another problem of ambiguity. For example, the SNP A123T could describe a NSM or a PSM. Several rules to disambiguate PSM and NSM are described in [35]. An approach for this disambiguation based on machine learning techniques is described in [60].

### Miscellaneous pitfalls

Additionally to the aforementioned problems, pitfalls that are obvious but not neglectable are reported: 1.) Some SNPs may not be contained in the SNP database of interest. This may have several reasons like a missing submission or a rather low minor allele frequency. 2.) SNPs reported in non coding regions are difficult to normalize as the current human mutation nomenclature covers these only in terms of genomic descriptions. Nevertheless, publications often describe these SNPs in relation to the closest gene, which is not covered in dbSNP. 3.) Not all needed information might be explicitly mentioned in the abstract. For example, the associated gene might be only mentioned in the full text article. 4.) Typos can basically appear in all types of descriptions (alleles, locations,…) and may make the normalization infeasible.

## Previous approaches for SNP extraction

Manual extraction and normalization of SNP mentions is time consuming but feasible for specific domain topics. For example, the AlzGene database [61] contains manually harvested information about SNPs from full text publications associated with Alzheimer’s disease.

Collecting such information on large scale requires automated methods due to the large amounts of published literature. Several text mining approaches for different purposes have been developed in the Life Science domain [62,63]. A typical problem for proper identification of biological entities in text is the lack of a common and widely established nomenclature. In context with the human variation nomenclature, many different ideas have been discussed [31,32,36-39,64]. In comparison to other NER tasks, only a few publications concentrated on the identification of SNPs and other types of small sequence variations in full text publications.

### Horn et al. 2004

MuteXt [33] is an early method for the extraction of single point polymorphisms. MuteXt uses regular expressions to extract protein names and variation mentions. The system performance has been estimated for the protein families “nuclear hormone receptors” (NR) and “G-protein coupled receptors” (GPCR) and achieves a precision of 87.9 % and 85.8 % and a recall of 49.3 % and 64.5 % for GPCR and NR respectively.

### Rebholz-Schuhmann et al. 2004

The tool MEMA [29] also uses regular expressions to extract variation mentions and gene names. In contrast to MuteXt, the system extracts variations on both nucleotide and amino acid level using the HUGO nomenclature [65] to automatically compile a dictionary to extract gene names. On a validation set consisting of 100 randomly selected MEDLINE abstracts containing either the key word “mutation” or “polymorphism” the system achieves a precision of 75% and a recall of 98%.

### Caporaso et al. 2007

An additional tool, solely concentrating on the extraction of non-synonymous variation mentions, is the freely available application MutationFinder [52]. Nonsynonymous mutations are a special type of SNP, because they alter the encoded polypeptide chain and are therefore often described on amino acid level. The authors created a set of 759 patterns, to cover the most recent descriptions of variation mentions in text. On a published validation set consisting of 508 abstracts with 910 variations the system achieves a precision of 98 % and a recall of 81 %.

### Yip et al. 2007

An alternative approach described by Yip et al. [34] focuses on the enrichment of sequence variations in the modSNP database [66]. Similarly to the previous approaches, non-synonymous mentions are extracted using regular expressions. Extracted variations are associated with the respective protein, allowing for validation of the extracted wild type amino acid with the amino acid contained in the corresponding UniProt entry. The authors describe rules to handle systematic errors based on liberties in numbering of protein variation mentions. This information includes evidence for post-translational cleavage or alternative splicing, which may result in different sequence length and therefore in different sequence numbering. The authors report that using the annotation information covered in UniProt allows to validate about 20 % more variation mentions. The system achieves a precision of 89 % and recall of 84 % on the validation corpus provided by MutationFinder.

### Furlong et al. 2008

A different approach is OSIRIS V1.2 [53], which identifies and normalizes any type of SNP (coding or non coding) to dbSNP identifiers. After selecting the genes mentioned in the abstract, the system retrieves all SNPs located on these genes and their corresponding terminology according to a SNP thesaurus. The terms are used for a pattern based search in the text and if found, the variation mentions are normalized to their corresponding database identifiers. The system achieves a precision of 99 % with a recall of 82 % on a validation set of 105 articles.

### McDonald et al. 2004 and Klinger et al. 2007

The usability of conditional random fields [67] to extract variation mentions, has been demonstrated by two approaches [43,57]. The latter approach also extracts variation mentions described in the latest human mutation nomenclature and direct mentions of dbSNP identifier by regular expressions. For the extraction of protein and gene names, the rule and dictionary based approach ProMiner [49] is used. A normalization module maps identified variations to an unambiguous dbSNP identifier based on the extracted entities. On a corpus of 105 abstracts the normalization achieves a precision of 78 % and recall of 67 %.

### Rhee et al. 2008

While regular expression-based recognition of dbSNP identifiers is a component of the previously mentioned system, the approach medRefSNP [68] focuses only on the extraction of such mentions from MEDLINE articles and OMIM. For this purpose, medRefSNP retrieves a list of relevant articles and extracts all dbSNP identifiers. The same is performed for all OMIM entries containing “Allelic Variants”. Further information about extracted SNPs is retrieved from dbSNP. Data on linkage disequilibrium is downloaded from HapMap and cytoband location from the UCSC genome browser [69]. SNPs located on genes are mapped to their specific Entrez Gene identifier. If available, pathway data for the gene is retrieved from KEGG [70]. One disadvantage of the described approach is that only direct rs mentions are found in articles. Variations described only in terms of natural language cannot be found.

### Krallinger et al. 2008

An approach to extract human kinase mutations is presented by Krallinger et al. [35]. Mutation mentions are extracted using MutationFinder and are classified into the categories natural- or induced-variant using a support vector machine classifier. The authors describe several simple but elegant rules to categorize ambiguous mutation mentions into PSM or NSM. For instance, 99.25 % of all PSM annotated in UniProt have a position number below 4000. Subsequently PSM are validated by using sequence information of the associated protein. Systematic errors in protein sequence numbering are also handled by different strategies.

### Recapitulation

All discussed approaches are able to identify variation mentions in scientific texts. Although a framework for the systematic analysis of mutation extraction systems exists [71], the results of the described systems are, due to the different foci, barely comparable. Some systems like MuteXt, its successor Mutation GraB [58], the approach published by Yip et al., Krallinger et al. and Laurila et al. [72] validate extracted PSMs by comparing the wild type amino acid with the amino acid stored in the corresponding UniProt entry. These articles describe observed problems concerning different residue numbering between the article and the protein sequence in UniProt. However, association of PSMs with protein identifiers is ambiguous, as different PSM may be associated with the same protein. Furthermore, such approaches neglect the normalization of NSM mentions, which provide a much higher ambiguity than PSM and are, therefore, more difficult to normalize.

Only OSIRIS and our approach associate extracted variations with unique SNP identifiers. Prior to the normalization of variations mentions with dbSNP, these approaches associate variation mentions with the corresponding gene or protein identifier. Therefore, these two approaches not only provide a normalization of variation mentions to dbSNP but also an association of variation mention to the corresponding gene/protein.

## Methods

### Corpus generation

To find and describe typical real world problems, a corpus consisting of SNP mentions associated with their dbSNP accession number mentioned in the text has been generated. Only few articles describe a variation in terms of natural language and the corresponding rs number. An initial list of 2,232 relevant articles has been received from dbSNP help desk. These abstracts were annotated by dbSNP and are known to mention at least one dbSNP identifier. The 2,232 abstracts were then automatically screened for SNP mentions using a modified version of MutationFinder.

Modifications encompass five regular expressions matching different NSM variants of the notations introduced in Section *Human Mutation Nomenclatures*. These modifications allow the detection of NSM mentions and are available in the supplementary material. The strict amino-acid alphabet of MutationFinder has been expanded to match ambiguous symbols like Xle, which can be used to describe the two amino acids leucine or isoleucine. Also different variations of termination symbols like term, amber or opal have been added to detect nonsense mutations. Additionally, regular expressions matching variation mentions using the latest recommendations for a human mutation nomenclature have been generated. The mutation mentions described on the homepage of the human genome variation society have been used for developing these regular expressions.

All regular expressions have been applied to the initial corpus of 2,232 articles. Extracted SNP mentions are then manually checked and associated with the corresponding dbSNP entry. SNPs missed by any of the regular expressions are also added into the corpus, if they could be associated with a dbSNP identifier. Identical descriptions were extracted only once per abstract. This procedure resulted in 527 variation/rs number pairs. From the 385 distinct rs numbers, 21 were found to use outdated dbSNP identifiers. These were replaced by the currently valid identifiers of dbSNP build 128.

The main properties of our corpus are as follows:

• The 527 SNP mentions can be separated into 283 PSM and 244 NSM.

• 48 SNP mentions refer explicitly to the type of used reference sequence

• 19 SNP mentions use the IVS concept to describe intronic variations

• 17 PSM are ambiguous as they could be potentially interpreted as NSM

### Implementation details

For each rs number we extract information about the associated gene, the position on the chromosome, known alleles, and the orientation of the SNP in relation to the associated gene. This information is extracted from a local copy of dbSNP. If the SNP is located in the coding region, the respective amino acid residues are also extracted from dbSNP. To allow for compensation of systematic differences in numbering, we follow the approach described by Yip et al. and parse information about post translational modifications from UniProtKB. Gene centric information, like exon/intron boundaries or the location of the start codon is extracted from Entrez Gene and ENSEMBL databases. Although both databases use the same genomic reference sequence, the boundaries for some transcripts (and their number) differ. Therefore, information about transcripts has been included from both databases. Gene name recognition and normalization to Entrez Gene and UniProt is performed using ProMiner. For evaluation we assume a perfect named entity recognition of gene names, by manually adding genes missed by ProMiner but required for subsequent SNP-normalization. This information can be gathered directly from the corpus, as dbSNP entries are associated with their corresponding Entrez Gene identifier. This allows a realistic assessment of the normalization procedure as the normalization algorithm is not influenced by the limited recall of a gene name recognition procedure. Subsequently, the algorithm retrieves for each SNP mention in the corpus a list of dbSNP candidates. The list of candidates is collected by retrieving all dbSNP entries associated with a gene contained in the article. In other words the SNP mention is associated with all genes mentioned in an article. The method iterates over all dbSNP candidates and performs a validation for the specific SNP mention.

**Figure 9:**
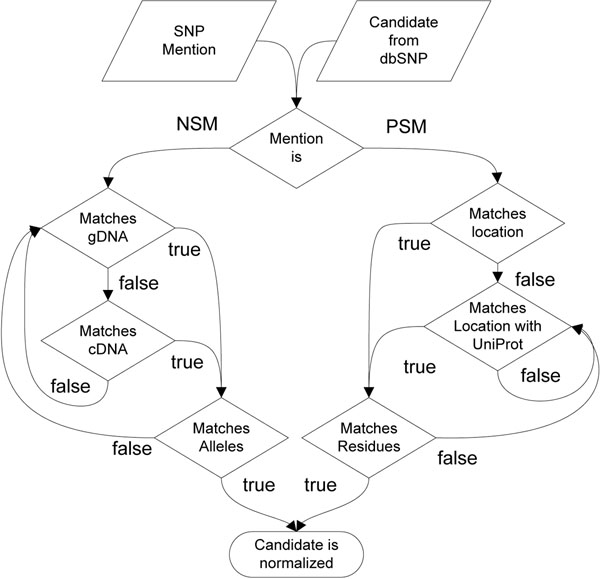
Flow chart of the normalization procedure.

The normalization algorithm for one SNP mention and one dbSNP candidate is depicted in Figure 9. The workflow disambiguates between PSM and NSM mentions. For ambiguous mentions like A123T the algorithm pursues both normalization strategies:

1.) For normalization of PSM the method first matches the extracted position against the dbSNP candidate. In case of a match the residues of the SNP mention are compared against the dbSNP residues. If both residues of the SNP mention are contained in the dbSNP candidate, the algorithm normalizes the mention. If the location of the SNP mention can not be validated, the algorithm incorporates knowledge about post translational modifications from UniProtKB. If any post translation modifications explains the difference in numbering the residues are validated. In case of a match the SNP mention is associated with the candidate. Otherwise the candidate is discarded.

2.) For NSM normalization the normalization procedure is more sophisticated, as several counting variants have been introduced. For each dbSNP entry we rebuild all different counting variants as described in Section *Human Mutation Nomenclatures* depending on the information where the SNP is located (Exon, Intron, UTR). These counting variants are calculated for each reference sequence associated with the current candidate entry. The algorithm reverses alleles (e. g. A– >G becomes T– >C), if the dbSNP reference sequence is located on the opposite strand than the reference sequence. Again, the algorithm attempts to validate first the position of the SNP mention with the dbSNP candidate. If the location of the SNP mention complies with any of the counting variants the corresponding alleles are also compared.

The algorithm does not distinguish between wildtype and mutated allele/residue as this information is usually based on the frequency in the observed population sample and does not necessarily resemble the recommendations for a human mutation nomenclature. Therefore, the normalization procedure checks if both alleles/residue are at the correct location, but allows changes in the directionality (e. g. A– >G becomes G– >A).

## Results and discussion

The general workflow described in Section *Normalization Process* was implemented as mentioned in Section *Implementation details*. The implementation correctly found 356 out of 527 variation/dbSNP pairs. The recall is 67.5% with a precision of 98.1 % (7 false positives in total). These 356 true positive SNPs can be further divided into 268 PSM and 88 NSM. Therefore, the recall is 94.7 % and 36.0 % for PSM and NSM respectively. Based on a manual inspection of each variation and the challenges described in the previous sections, typical problems and pitfalls are highlighted in the following.

### Inspection of normalization issues

#### Protein sequence mutations

As previously described, two different counting variants for PSM are known. First, three PSMs with one digit lower than in the dbSNP entry have been found in the corpus. The reason for the different numbering is that the initiator amino acid is cleaved from the primary translation product. This leads to the observed difference of 1 between the textual description and the dbSNP entry. The corresponding authors were contacted and affirmed the deprecated property of the stated description. The authors used this description, because it is a long established term in the community. For all PSMs this information is covered in the UniProt feature *initiator methionine*. More information about these specific mutations is described in Table 3.

**Table 3:** Normalized SNPs which derive the location one position after the leading amino acid.

PMID	dbSNP identifier	Mention in text	Mention in dbSNP
16489054	rs605059	Ser312Gly	Ser313Gly
16525568	rs5985	Val34Leu	Val35Leu
17241179	rs5985	Val34Leu	Val35Leu

Second, eight PSMs can be recovered using the “sequence annotation” feature information from UniProtKB. A complete list of recovered SNPs is depicted in Table 4. It shows which PSM could be recovered by using which UniProtKB entry and feature. It is noteworthy that not all PSMs could be recovered using UniProt as additional information source. For example, Ha et al. [73] describe three different non synonymous SNPs on gene SDC3. For all three SNPs a constant difference of 52 amino acids in comparison to the described dbSNP entry can be observed. However, no feature of the corresponding UniProtKB entry (O75056) provides evidence for this constant offset. At present it is unclear to us from which reference sequence these positions have been derived. These PSMs could have been normalized using the methods proposed in [35,72] to handle systematic numbering errors for PSM.

**Table 4:** Overview of all recovered polymorphisms using UniProtKB as additional data source.

PMID	dbSNP identifier	Variation	UniProtKB id	Used feature
16368448	rs5063	Val7Met	P01160	Signal: 1-25
17196207	rs5882	I405V	P11597	Signal: 1-17
17344938	rs1123617	Val227Ile	Q15849	Var_Seq: 1-523
17344938	rs3745009	Ala357Thr	Q15849	Var_Seq: 1-523
17517687	rs1041981	Thr26Asn	P01374	Signal: 1-34
17634448	rs2230199	Arg80Gly	P01024	Signal: 1-22
17944986	rs6136	Thr715Pro	P16109	Signal: 1-41
18034366	rs5882	I405V	P11597	Signal: 1-17

The fourth column refers to the used UniProtKB entry and the fifth column shows which specific feature has been used to recover this variation.

#### Location of alleles on opposite strand side

As previously described, the dbSNP reference sequence can be located on the opposite strand than in the mentioned NSM. This property is observed seven times in the dataset. Therefore, the dbSNP entry describes the alleles on the opposite strand than the variation mention in the text. To successfully recover those variations one has to reverse the alleles contained in the dbSNP database. Detailed information for these NSMs is listed in Table 5.

**Table 5:** Normalized SNP mentions, where the alleles of dbSNP (build 128) are located on the opposite strand than the corresponding gene.

PMID	dbSNP identifier	Variation	Alleles in dbSNP
16144952	rs2077647	T30C	A/G
17480010	rs1867561	–135C→G	C/G
17495420	rs1572983	59G→A	C/T
17630229	rs5569	G1287A	C/T
17917281	rs5569	G1287A	C/T
18203168	rs234706	C699T	A/G
18280297	rs4614723	3823G→A	C/T

#### Permutation of dbSNP identifiers

Additionally to these systematic errors, the numeric patterns of five rs numbers are wrongly described in the text. A detailed description can be found in Table 6. It can be seen, that the rs numbers for three SNPs differ only in one digit and for the remaining two entries two digits have been transposed. It is noteworthy that the rs number of the first variation “V660L” is described once correctly and once wrongly in the abstract. It is therefore probable that the wrong description occurred due to a transcription error. None of the remaining dbSNP entries represent the described variation in the text. Two variations (rs8192673 and rs861529) are located in intronic regions and one (rs2308237) is located on genomic background, where the described non synonymous mutations cannot occur. For rs8192673 the error has been discovered and an erratum has been published [74]. Non-systematic errors like these may occur in different steps of the publication process. It is likely that similar errors also happen for other numbers like the location of an mutation or accession numbers. However, such errors should be rare and we have not found such errors in our corpus.

**Table 6:** List of SNPs described by false dbSNP identifiers in the publication.

PMID	Variation	Specified dbSNP identifier	Correct dbSNP identifier
16614108	V660L	rs1042638	rs1042**8**38
17301261	1793G>A	rs2274976	rs22749**67**
17390150	G482S	rs8192673	rs819267**8**
17701750	T241M	rs861529	rs8615**3**9
18268114	K178R	rs2308237	rs2308**32**7

The observed difference between correct and wrong identifier is highlighted in bold and usually is one or two digits.

Independent from our corpus an error in the description of a SNP by Yoneyama et al. [75] has been observed. The authors describe the substitution of an alanine to proline at amino acid 459 in COL1A2. We believe that the correct location is amino acid 549 and the corresponding dbSNP identifier is rs42524. A first indicator is that at position 459 an isoleucine is located instead of the described alanine and no UniProtKB feature provides evidence for a systematic error. In the corresponding letter by Arnold et al. [76] the variation is described as Ala549Pro (rs42524).

#### Duplicate dbSNP entries

Due to the building process of dbSNP more than one rs entry for one SNP can be generated. This is the case when the flanking region of the ss entries substantially differs. In this case the genomic location for both dbSNP entries is the same but the dbSNP entries are not merged. This has been observed for two SNP mentions, which could be associated to both rs numbers. The entries are shown in Table 7. Please note that the second mention has been already merged to rs6670 in the latest dbSNP release.

**Table 7:** SNPs with more than one valid dbSNP entry.

PMID	SNP	rs number in text	also valid dbSNP identifier
16652158	–77T>C	rs11553656	rs3213245
17289909	A8618T	rs45566835	rs6670

#### dbSNP entries associated with no gene entry

During the analysis we observed that five dbSNP entries are not associated with the gene mentioned in the text. According to dbSNP the entries shown in Table 8, are not associated with any gene. This is reasonable as all mentions describe a variation located far upstream in the promoter region of the gene mentioned in the text. To normalize such mentions one would have to extract the genomic coordinates of a gene and find dbSNP entries approximate to this gene.

**Table 8:** SNPs without gene association according to dbSNP.

PMID	SNP mention	dbSNP identifier	Entrez Gene Identifier
15823203	–3608T>C	rs7379701	9607
16670163	g.-420C–>G	rs862513	56729
17363416	c.-9610G>A	rs8007267	2643
17604842	-C8347G	rs4131347	121278
18059035	G-2548A	rs7799039	3952

The last column mentions the associated gene according to the original article.

#### Ambiguity between PSM and NSM

The short form of 17 PSM is ambiguous in that it could also be interpreted as a NSM. Some examples are provided in Table 9. All 17 mentions would have been correctly identified as PSM by the rules mentioned in [35]. It is noteworthy that this problem might be self-inflicted as some tools, like MutationFinder, normalize amino acids to one letter codes. For example the mention Ala357Thr would be normalized to A357T and the mention would have become ambiguous. Considering that information would have allowed to classify 9 mentions as PSM. Therefore this simple rule could be added to the ideas described by Krallinger et al. to distinguish PSM and NSM.

**Table 9:** Mentions of protein sequence mutations which might also refer to a nucleotide sequence mutation.

PMID	SNP	dbSNP identifier
16336637	A206T	rs2235491
16336637	G870A	rs603965
17096334	A394T	rs2305160
…	…	…

From 244 NSM, 65 use a minus character, 30 mention the used reference sequence (c. or g.), and 19 are described by the IVS concept. Therefore 114 of 244 NSM could be previously classified as NSM. For the remaining NSM the normalization algorithm performs a NSM and PSM normalization.

#### Change of directionality

The distinction between wild type and mutated residue/allele is usually based on frequency in the specific population sample. However, the human variation nomenclature advises authors to use the nucleotide/residue of the reference sequence as wild type. This sometimes leads to a change in directionality between the alleles/residues described in the mutation mention and the corresponding database entry. This problem can be observed for 86 out of 244 (35.2%) NSM and 53 out of 283 (18.7%) PSM in our corpus. Examples for flipped wildtype and mutated alleles are provided in Table 10.

**Table 10:** Examples of SNPs where a change of directionality between textual description and dbSNP entry can be observed.

PMID	SNP	dbSNP identifier
17582620	IVS3+411C>T	rs2486001
18300940	S312N	rs2293275
18470941	p.V432L	rs1056836
…	…	…

All problems observed in our corpus are summarized in Table 11

**Table 11:** Problematic cases contained in the corpus of 527 SNPs.

Occurrence	Type
3	Initial amino acid not counted
8	PSM deduced from mature protein
7	dbSNP entry on reverse strand
5	Typing error in rs number
2	Ambiguous dbSNP entries
5	Mutation not associated with the mentioned gene
17	Ambiguous PSM
139	Change of directionality

## Conclusions

Normalization and interpretation of SNP mentions is highly challenging for human readers as well as automated machineries. In this paper, we discussed several common pitfalls which have to be overcome for successful normalization of variation mentions to dbSNP. Most of them are generally valid and apply also for other SNP databases.

In particular we depicted a number of observed real world examples based on a corpus of 527 SNP/dbSNP pairs. Using this corpus reveals non-systematic errors like permutation of numbers, which are hard to find. We demonstrate that our developed normalization algorithm produces precise results for both PSM and NSM. However, the recall for the normalization of PSM is substantially better (94.7 %) than for NSM (36.0 %). We believe that this might have several reasons: First, our approach currently incorporates no provenance information and uses only the latest gene annotations from ENSEMBL and Entrez Gene. Second, the early papers on mutation nomenclature pointed out, that the lack of a complete reference sequence might lead to an almost arbitrary position numbering and domain experts might stick to these deprecated references. Third, manual conversion of a SNP into any mutation nomenclature is, without any computational assistance like Mutalyzer, error prone. Finally, sequencing errors might lead to small but substantial differences in numbering. To overcome the limited recall for NSM normalization we plan to incorporate RefSeq transcripts, which are derived from GenBank and provide current and deprecated annotations for genes.

We believe the developed corpus will help to facilitate further development in the normalization of SNPs to dbSNP identifiers and will assist the community progress toward a common corpus useful for the systematic evaluation of grounding tools. The annotated corpus is available at http://www.scai.fraunhofer.de/snp-normalization-corpus.html.

## List of abbreviations and recently used biological terms

• cDNA: Complementary DNA

• gDNA: Genomic DNA

• IVS: Intervening Sequence

• Mutation: Refers to rare variants which often cause diseases and affect conserved residues in the protein sequence. Also used to refer to modified residues in a sequence after the experimental procedure of mutagenesis; NSM: Nucleotide Sequence Mutation

• PSM: Protein Sequence Mutation; SNP: Single Nucleotide Polymorphisms or SNPs are DNA sequence variations in which a single nucleotide (A, G, C or T) is altered. SNPs are also referred as polymorphisms, natural variants, or common variants because they have a minor allele frequency in the population of at least 1 %. In contrast, rare variants have a minor allele frequency of less than 1 %. SNP mention: Textual description of a SNP

• UTR: Untranslated Region

• Variation: Any kind of short range sequence variation in the nucleotide sequence of the genome

• Variation mention: Textual description of a variation

## Competing interests

The authors declare that they have no competing interests.

## Authors contributions

PET and RK wrote the main parts of this paper. PET annotated and analyzed the corpus and implemented the system. RK, LIF and CMF were involved in substantial discussions of aspects described here and revised this contribution. MHA critically revised the manuscript.
